# Nanotechnology for research and treatment of the intestine

**DOI:** 10.1186/s12951-022-01517-3

**Published:** 2022-09-29

**Authors:** Yanquan Fei, Yong Ma, Huaizu Zhang, Hao Li, Guangfu Feng, Jun Fang

**Affiliations:** 1 College of Bioscience and Biotechnology, Hunan Agricultural University, Hunan Provincial Engineering Research Center of Applied Microbial Resources Development for Livestock and Poultry, Changsha, 410128 Hunan China; 2 School of Pharmaceutical Sciences (Shenzhen), Sun Yat-Sen University, Shenzhen, 518107 Guangdong China

**Keywords:** Nanotechnology, In vitro modeling, Intestinal disease, Precision treatment

## Abstract

The establishment of intestinal in vitro models is crucial for elucidating intestinal cell-microbe intrinsic connections and interaction mechanisms to advance normalized intestinal diagnosis and precision therapy. This review discusses the application of nanomaterials in mucosal therapy and mechanism research in combination with the study of nanoscaffold in vitro models of the gut. By reviewing the original properties of nanomaterials synthesized by different physicochemical principles and modifying the original properties, the contribution of nanomaterials to solving the problems of short survival period, low cell differentiation rate, and poor reduction ability in traditional intestinal models is explored. According to nanomaterials’ different diagnostic mediators and therapeutic targets, the current diagnostic principles in inflammatory bowel disease, intestinal cancer, and other diseases are summarized inductively. In addition, the mechanism of action of nanomedicines in repairing mucosa, inhibiting inflammation, and alleviating the disease process is also discussed. Through such systematic elaboration, it offers a basis for nanomaterials to help advance in vitro research on the intestine and provide precision treatments in the clinic.

## Introduction

The intestinal system is the primary site of nutrient absorption, and studies have shown that the intestinal system can also coordinate with the brain and other tissues to regulate homeostasis through the sensing of nutrients [1]. The occurrence of diseases such as intestinal inflammation seriously affects the homeostasis of the human body environment, causing great trouble to human life and health [2]. Since the 1990s, intestinal diseases, including inflammatory bowel disease and irritable bowel syndrome, have increased in newly industrialized nations in Africa, Asia, and South America, with Europe having the highest prevalence. The intestinal disease has become a worldwide public health concern. Complicated and expensive investments in preventive research and medical treatment have placed an enormous financial burden on communities [3, 4]. Most intestinal diseases like inflammatory bowel disease are hereditary, have a low cure rate, and require medication over the entire lifespan once diagnosed [5]. Even more alarming is that intestinal disease may rapidly advance to colon cancer, which has the second-highest mortality rate of all cancers, through an inflammation-mediated pathway [6]. Traditional medical methods can provide corresponding drugs and surgical treatments for the clinical symptoms of intestinal diseases. However, due to the complexity of the etiology of intestinal diseases and the uncertainty of the pathogenesis [7], the treatment outcomes are rarely sufficient [8, 9]. Moreover, the traditional methods of treating intestinal diseases currently have a number of shortcomings, including the low efficiency of targeted drug delivery, drug resistance caused by high doses of drugs, and drug side effects [10]. For inflammatory bowel diseases, anti-inflammatory drugs such as mesalazine, balsalazide, infliximab, and adalimumab are the first-line treatment options [11, 12]. After oral administration, these drugs are rapidly and widely absorbed in the digestive system, especially the proximal gastrointestinal tract, and rarely act on inflammation and lesions [13]. Meanwhile, these drugs can cause a series of side effects, such as diarrhea, nausea, abdominal pain, headache, vomiting, and rash when the dose increases [14]. For colorectal cancer, drug therapy such as intravenous chemotherapy also has problems, such as acquired drug resistance and off-target toxicity [15]. On the other hand, in vitro models for the study of gut disease mechanisms have limitations. Although more and more animal (mice, pigs, and nonhuman primates) models are being used to study the pathogenesis of diseases, the individual differences in species, biological environment, and other factors cause differences between animal model mechanisms and clinical trial studies [16]. In addition to animal models, most in vitro models applied to intestinal diseases are two-dimensional cell models. This research mode simplifies the complex internal environment of the organism, focuses only on specific cellular responses, and lacks consideration of the human body, intestinal tissue, and intestinal homeostasis among microorganisms [17].

Given the shortcomings of traditional medical and research methods, nanomaterials seem to offer an excellent potential solution. Nanomaterials have received increasing attention in the life sciences due to their tiny structure (1–100 nm in size), large surface-to-volume ratio, and biocompatibility [18]. Nanoscale-size materials are able to pass more readily through the vascular epithelium to the tissue lesion site owing to their enhanced permeability and retention (EPR) effect [19]. Additionally, the outstanding specific surface area provides a variety of loading sites or reactive sites for modification and alteration [20]. For instance, some nanomaterials exhibit well-stabilised catalytic enzyme activity and broad-spectrum scavenging capacity against hazardous reactive oxygen species [21], as well as excellent stability and quick scavenging in harsh disease environments [22]. In addition, many antibacterial nanoparticles (NPs) can be ingested by pathogenic bacteria, destroying protein structure and causing DNA damage in the bacterium for antibacterial and anti-inflammatory effects [23]. Moreover, nanomaterials with targeting properties are widely considered for drug transport systems. As drug delivery systems, nanomaterials provide targeted and continuous drug release for diseases and enhance therapeutic efficacy due to the controlled drug release and improved pharmacokinetics and pharmacodynamics, ensuring higher drug bioavailability, longer duration, and minimal toxicity relative to conventional therapies [24]. Meanwhile, the nano delivery systems of peptides, nucleic acids, and genes transfect immune cells in vivo without causing toxicity or unwanted immunogenicity, gradually becoming essential in cancer therapy to meet current medical needs [25]. Furthermore, nanoparticles possess superior mechanical and morphological qualities and have been extensively tested as nanoscaffolds in organ tissue engineering. They can facilitate cell adhesion and proliferation in nerve [26], bone [27], skin tissue [26], and are substitutable [28]. Specifically, the high porosity, interconnectivity, mechanical strength, and high biocompatibility of nano scaffolds can offer an optimal porous network for cell growth, which promotes cell adhesion, proliferation, and differentiation and enables precise replacement of damaged tissues in tissue regeneration [29]. For example, a 3D co-culture of nanohydrogels with an extracellular matrix (ECM) can form villi-like, crypt-like and tubular scaffolds in vitro, remodelling the complex structure of intestinal tissue [30]. Because of the complex composition of the internal environment in vivo and the close connections between multiple cells and tissues, simple in vitro cell cultures do not fully reflect the study results [31]. However, this scaffold also facilitates in vitro studies, and with the help of nanoscaffolds, re-establishing such connections in vitro also helps to make the results more convincing.

**Fig. 1:**
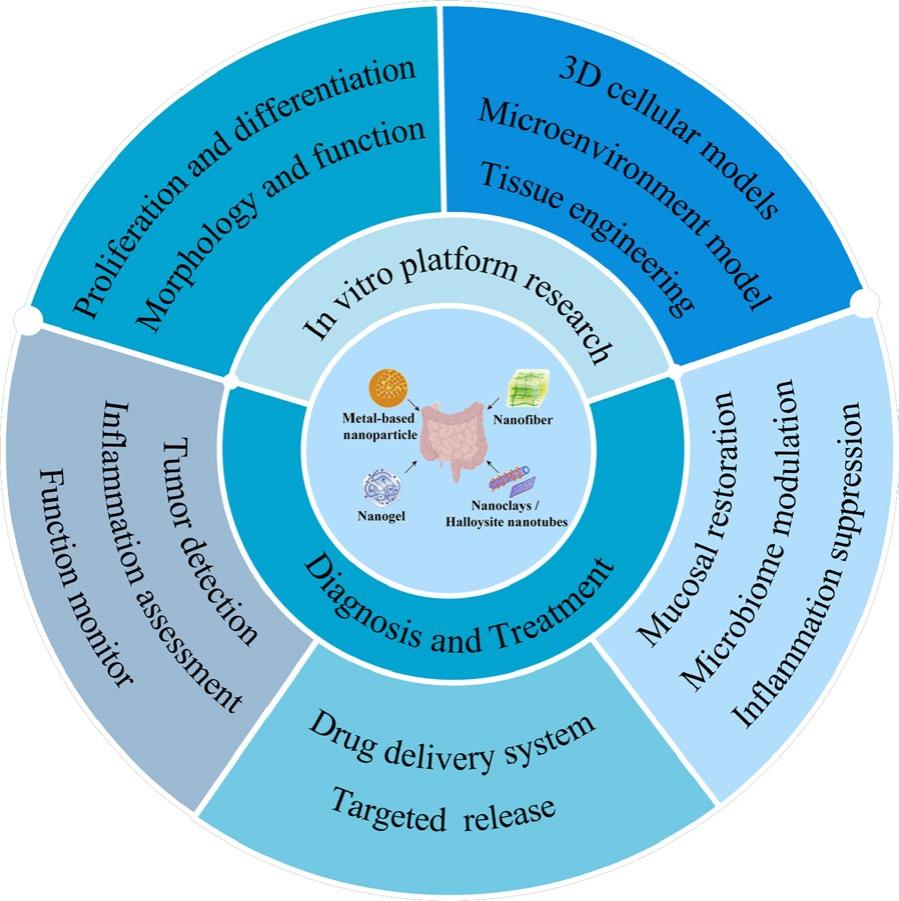
Application of nanomaterials in intestinal health

Herein, we discuss the application of nanomedicine in mucosal therapy and mechanism research in combination with the study of nanoscaffold in vitro models of the gut. First, from the perspective of nanomaterial-assisted research, recently reported intestinal research is systematically summarised, and the application of four classical nanomaterials to the establishment of in vitro intestinal models and to the progress of disease diagnosis and treatment is shown (Fig. 1). The nanomaterials synthesised on the basis of different physicochemical principles solve the problems of short survival period, low cell differentiation rate, and low reduction of traditional intestinal models by changing the original properties. This makes the nanoscaffolds more competent for intestinal-related research in a physiological environment. Second, concerning the nanomaterials’ different diagnostic mediators and therapeutic targets, the current diagnostic principles in inflammatory bowel disease, intestinal cancer and other diseases are summarised, and the mechanism of mucosa repair, inflammation inhibition and alleviation of the disease process are surveyed.

## Types of nanomaterials applied to intestinal health

**Fig. 2:**
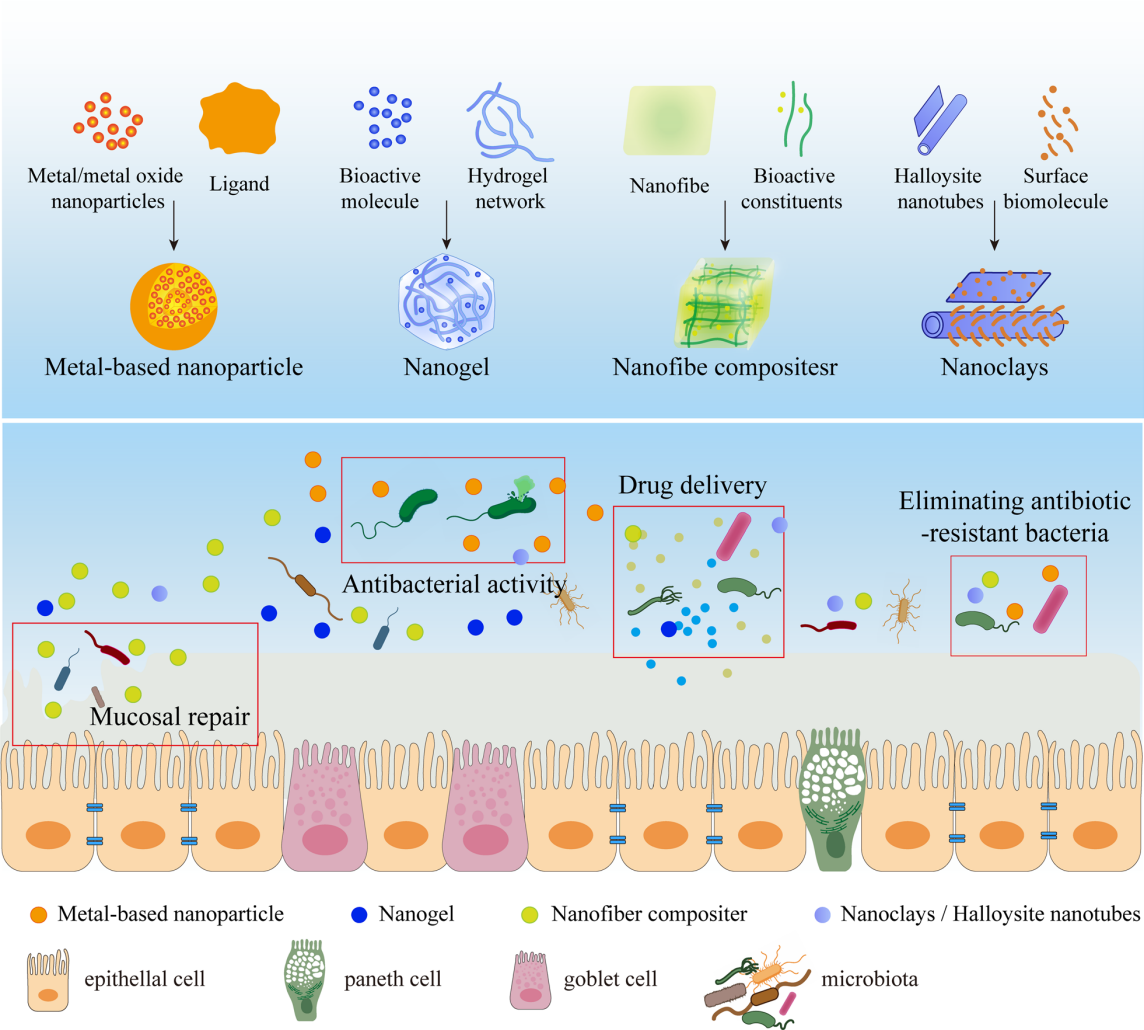
Synthesis of different types of nanomaterials and their influences on the intestine. **A** Brief schematic diagram of the synthesis methods of the four nanomaterials. **B** Several main functions of nanomaterials in intestinal microbiota and enterocytes

Nanomaterials have risen to prominence among numerous materials due to their superior performance, becoming the preferred option in many research fields. The health of the gastrointestinal system has received more attention in recent years since it directly impacts the body's immunity and metabolism. Nanomaterials play an incredible role in promoting and maintaining the health of the intestinal tract. The kinds of nanomaterials employed in gut health are discussed in this section (Table 1), as well as the synthesis methods and their various functions in the gut (Fig. 2).

**Table 1:** Nanomaterials related to the intestine

Type	Nanomaterials	Function	Refs.
Metal-based NPs	Ag NPs (plant-mediated)	Antibacterial activity	[32]
	AgPd_0.38_ nanocages	Eliminating antibiotic-resistant bacteria	[33]
	ZnO NPs	Antibacterial activity	[34]
	Cu NPs	Antibacterial activity	[35]
	Au NP-AMP	Eliminating antibiotic-resistant bacteria	[36]
	Au NPs	Medical imaging	[37]
Nanogel	Graphene oxide- quaternary ammonium salt hydrogels	Antibacterial activity	[38]
	Chitosan-montmorillonite hydrogels	Tissue engineering	[39]
	Polynitrogen-isopropylacrylamide and acrylic acid (pNIPAM-co- AAc) hydrogels	Drug delivery	[40]
	Pseudopeptides and decylamine (PLP-NDA) nanogel	Drug delivery	[41]
	Cyanidin-3-O-glucoside Nanogel	Chemical stability and Antioxidant activity	[42]
Nanofibers	Nanofiber film (fatty acid chloride)	Food packaging	[43]
	Amoxicillin trihydrate-Bombyx mori silk fibroin	Drug delivery	[44]
	Insulin-hydroxypropyl methylcellulose phthalate NPs	Drug delivery	[45]
	Cellulose nanocrystals -CaCl_2_	Drug delivery	[46]
Nanoclays	Chitosan-functionalized HNTs grafting copper and laccase	Enzymatic catalysis	[47]
	3-aminopropyltriethoxysilane functionalized HNTs (APTES-HNT)	Ionic adsorption	[48]
	Ciprofloxacin enclosed in APTES-HNT	Drug delivery	[49]
	Microparticles embedded in amine-functionalized HNTs	Drug delivery	[50]
	Paclitaxel encapsulated in HNTs	Drug delivery	[51]

### Metal-based nanoparticles

Metallic nanoparticles are usually made of metal oxides or inorganic substances to wrap the metalcore. They have outstanding features of chemical stability, broad-spectrum antibacterial, optical properties, and biocompatibility, commonly used in biosensing sickness treatment and optical imaging because of their properties [52]. Gold, silver, aluminum, iron, copper, and zinc are popular metals utilized to produce metal nanoparticles relevant to intestinal health.

#### Antibacterial efficacy modulating intestinal microbial homeostasis

Firstly, metal nanoparticles are frequently applied in treating intestinal pathogen infection due to their broad-spectrum antibacterial characteristics. For example, silver nanoparticles (AgNPs) obtained by mixing plant extracts and silver salt and then reductive treatment show antibacterial efficacy against *Escherichia coli* and *Micrococcus luteus*, two pathogenic intestinal pathogenic germs, with negligible cytotoxicity [32]. Because most harmful bacteria include Ag^+^-binding proteins, AgNPs can perform antibacterial functions owing to decrease enzyme function by binding His185 residue. It can also induce reactive oxygen species (ROS) accumulation to cause bacterial death [53]. AgNPs and potassium chloropalladite synthesize AgPd_0.38_ nanocages by electrical substitution reaction, an oxidase model. The surface-bound nature and endocytosis of ROS generated by this nanozyme exist unexpected detoxification that effectively eliminates antibiotic-resistant bacteria and delays the emergence of bacterial resistance [33]. Except for argentum, copper has also been shown to exhibit antibacterial activity against both Gram-positive and Gram-negative bacteria. Not only can Zn NPs directly interact with the phospholipid bilayer of cell membranes, resulting in the loss of membrane integrity to be bactericidal, but they can also participate in the creation of ROS that impede bacterial survival [34]. In addition, copper-containing nanoparticles have also been shown antibacterial activity against *Saccharomyces cerevisiae*, *Escherichia coli*, *S. aureus*, and *Listeria monocytogenes *[35]. Because copper NPs can interact with amino and carboxyl groups on the surface of microbial cells, when Cu^2+^ ions are present in high concentrations, they will further induce the formation of ROS inside bacterial cells and thus ultimately inhibit the synthesis of pathogenic bacteria’s amino acids and the replication of genetic information [54]. Furthermore, alumina-containing nanoparticles also show the ability to destroy the cell walls of pathogenic microorganisms such as *Escherichia coli* and *Salmonella *[55]. According to relevant studies, Al_2_O_3_ NPs increased the expression of the bacterial cell membrane binding gene RP4, hence promoting the spread of antibiotic resistance [56]. Metal nanoparticles have also been utilized as pharmacological adjuvants to enter the body and exert therapeutic effects in the intestinal system, perhaps impairing the function of digestive juices. Additionally, metals' antibacterial properties may influence intestinal microbial homeostasis [57]. Thus, in the long run, it may be worthwhile that circumvents the enzyme-inhibiting impact of metal nanoparticles and instead leverages their antibacterial characteristics to aid in the restoration of intestinal microbial balance.

#### Optical properties assisting intestinal diagnosis

Besides, metal nanoparticles also exhibit the optical properties of surface plasmons [58]. This optical property has contributed significantly to its dominance in medical imaging and disease diagnosis [59]. Widely used photothermal therapies merged with monotherapies that utilize NPs in combination with surface markers of cancer stem cells can help develop new targeted diagnostic strategies [60]. Various metal nanoparticles have been developed to label and diagnose intestinal health research. At present, the most used metal nanoparticles in intestinal research are gold nanoparticles, which can be utilized as a luminous basis to label blood vessels in the colon and then using a 3D confocal microscope images the microscopic environment of the intestine and vasculature [37]. The triangular nanosensor synthesized with gold particles and polyaniline can be used to precisely detect a variety of intestinal functional parameters, including pH, contraction frequency, and amplitude, providing a new technology for clinical intestinal diagnosis [61].

#### Metal NPs delivery system involved in the treatment of intestinal diseases

Resistance to antibiotic therapy seriously affects the disease treatment process and the ecological dysregulation of the intestinal microbiota [62]. Based on the vast difference between the antibacterial mechanism of the metal nanoparticle delivery system and antibiotics, the emergence of microbial resistance can be fundamentally eliminated, whereas the synergistic treatment of metal-based NPs with antibiotics can effectively reduce the possibility of antibiotic resistance as well as the dosage employed [63]. To combat pathogenic microorganisms' medication resistance, studies have found that gold-containing nanoparticles combined with ampicillin (Au NP-AMP) can kill resistant bacteria such as *P. aeruginosa*, *Enterobacter aerogenes*, and *Escherichia coli* K-12 substrain DH5-*α*. Au NP-AMP provides an excellent example of synergistic therapeutic sterilization of nanoparticles and antibiotics at the lesion site. On the one hand, the ampicillin molecule bounds to the surface of Au NPs can inhibit bacterial expressing of *β-*lactamase. On the other hand, ampicillin transports Au NPs into the microbial cell membrane, where it exerts its antibacterial effect [36]. The synergistic antibacterial mechanism of NPs and antibiotics may be that metal-based NPs disrupt the cell membrane components of harmful bacteria to increase the permeability of the cell wall and promote the effective entry of antibiotics into the cells to exert bactericidal effects [64].

### Nanogel

Nanogel is a group of extremely hydrophilic, three-dimensional network gels with high water-holding capacity and good structural integrity, which can absorb different volumes of water and maintain three-dimensional structures to improve permeability for tissue regeneration, attracting widespread attention [65]. Hydrogel systems are superior versatility and load various materials as desired application functions through controlled chemical composition and cross-linking ratios [66].

Highly porous and hydrated network of the nanogel supports the delivery and release of various bioactive molecules. The addition of nanoparticles vastly enhance the mechanical strength of hydrogels [67]. For example, oleic acid vesicles load with 2, 3, 5, 4’-tetrahydroxystilbene 2-O-*β*-D-glucoside (THSG) are ionically cross-linked to the gel. After transdermal administration, the swellable diffusion of the composite gel greatly enhance the skin permeability of THSG [68]. Nanoparticles modified with biomolecules can also endue specific biochemical properties of thermal, electrical, optical, and magnetic to hydrogels [69]. Thus, hydrogel nanocomposites are highly recognized in the fields of diagnosis and treatment of intestinal diseases. With the development of tissue engineering and biomedicine, higher requirements are imposed on the performance of hydrogels. Besides stronger mechanical properties, antimicrobial efficiency is gaining significant attention. The most widely used method is to add metal nanoparticles with antimicrobial activity (e.g., Ag, Cu, ZnO_2_, TiO_2_ NPs) into the hydrogel network to overcome microbial drug resistance [70]. Additionally, graphene oxide sheets integrated with quaternary ammonium salt-based hydrogel matrix, demonstrated efficient antimicrobial and degradable, photochemical energy conversion, and intrinsic bactericidal activity [38]. To improve the biocompatibility of the hydrogels, a pharmaceutical product additive montmorillonite (MMT) (weakly negatively charged), is well mixed with methacrylated glycol chitosan (positively charged) polymer. The monolayer of MMT is evenly distributed within the solidified nanocomposite hydrogel, forming interconnected microporous structures. Interlay-pores and inter-particle spaces resulting from that allow cell migration in the environment and promote the interaction between cells and scaffolds, as well as cell proliferation and differentiation [39]. It has shown enormous potential and utility in the manufacture of biocarriers for stem cells, bioactive agents, or regenerative medicine tissue grafts.

### Nanogel carrier to enrich intestinal therapies methods

Through encapsulation, covalent and non-covalent interactions, the drugs can be packed in nanogels. After reaching the site of action, the drug is diffused or dissociated by swelling properties to achieve the therapeutic purpose [71]. The cross-linked structure of nanogels can keep the activity of large molecules such as proteins, oligonucleotides, peptides, and prevent their exposure and inactivation at non-target locations [72]. Most of the nanogels used in the intestinal delivery of drugs are the pH-induced release of drugs, and oral nanogels drugs undergo significant pH changes during the process from the stomach (pH = 2) to the intestines [73]. Based on this feature, pNIPAM-co-AAc nanogel is synthesized by copolymerizing polynitrogen-isopropylacrylamide and acrylic acid (pNIPAM-co-AAc). The *β*-lapachone loaded in pNIPAM-co-AAc nanogel has a heat-responsive and pH-responsive structure, which can be accurately positioned to the end of the small intestine, and play a pivotal role in the treatment of colon cancer [40]. It has also been shown that PLP-NDA nanogel carriers are formed using alkyl-linked branched pseudopeptides and decylamine (PLP-NDA) through physical cross-linking. The intestinal cell membrane is unstable under a slightly acid environment (typical intestinal pH: 5.0–6.0) and thus has highly excellent membrane permeability to facilitate the transmembrane transport of PLP-NDA nanogel drug macromolecules in intestinal epithelial cells [41]. Nanogels can also ensure the stability of active substances and improve their bioavailability even in acidic environments. Combining a maillard reaction with thermal gelation to produce a nanogel containing ovalbumin, dextran, and pectin, for instance, increases the resilience of the loaded protein to severe conditions and improves its practicality. At the same time, after this nanogel encapsulates cyanidin-3-O-glucoside, the electrostatic interaction between the flavonoid cation and the carboxyl group on the pectin enhances the hydrophilicity and resistance to pepsin hydrolysis [42]. In addition, the microfluidic preparation of gelatin methacrylate (GM) permits flexible microgel size variation to efficiently extend drug release time [74].

### Nanofiber composites

Nanofiber composites are highly crystalline needle-like cellulose nanostructures with a width of 10 to 20 nm and a length of several hundred nanometers. They have a high surface area to volume ratio, high tensile strength, high stiffness, and a high degree of flexibility [75]. Hydrolysis of natural cellulose with strong acids and alkalis is followed by the formation of nanofiber composites by high temperature, high pressure, homogenization, grinding, or microfluidization [76]. Moreover, the functional groups on the surface of nanofibers allow various surface modifications through a variety of chemical reactions, including esterification, etherification, oxidation, amidation, carbamate, acid hydrolysis, and nucleophilic substitution to obtain properties suitable for high-end technology and biological applications [77]. It is seen that nanofiber composites have tremendous application potential in tissue engineering due to their cost-effectiveness, sustainability, environmental friendliness, biocompatibility, biodegradability, and non-toxicity [78].

#### Modified nanofibers for targeted drug release

Nanofibers with modified surface properties can be used in antibacterial, intestinal precision nutrition, and targeted medicinal fields. Increased hydrophobicity and reduced mechanical characteristics of the film surface result from painting fatty acid chloride thinly over the nanofiber film to encourage the esterification process, which is beneficial for its application in food packaging [43]. Cold atmospheric pressure plasma surface modification can be accomplished for nanofiber polymers [79]. The oxygenated functional groups produced by using oxygen dielectric barrier discharge plasma are bound to the surface of the Silk fibroin (loaded with amoxicillin)/ polyvinyl alcohol (PVA) nanofibers, which increases the roughness and expands the contact area to promote the PVA degradation, rapid drug release, and enhances the antibacterial properties [44]. Meanwhile, surface modification can also greatly enhance the biocompatibility of nanofibers. Hydroxypropyl methylcellulose phthalate (HPMCP) NPs, a biocompatible intestinal material, is synthesized through a modified spontaneous emulsion solvent diffusion process [45]. Concretely, dilute hydrochloric acid containing insulin (target drug) is mixed with acetone containing HPMCP, then stirred and emulsified in PVA aqueous solution at 100–600 rpm before freeze-drying to obtain a product. This modified nanofiber material delivery system exhibits enhanced intestinal absorption. Also, a dual droplet-based freeform 3D printing (DDF3) method is developed. Sodium alginate is dissolved in direct ink writing at a concentration of 1–10% (w/v) and shaken for 10 h until it becomes transparent to obtain an alginate ink. Then, cellulose nanocrystals and 20% CaCl_2_ at 9:1 (v/v) are cross-linked in the alginate biomaterial inks to obtain a printing material. The experimental results show excellent shape fidelity and structural stability, with high viscosity in the range of optimal rheological properties to obtain a controlled and sustained release in the small intestine [46].

### Nanoclays/Halloysite nanotubes

Nanoclays are abundant in nature, have a high level of biocompatibility and sustainability, and have garnered considerable interest in the life sciences. Nanoclays, basically bentonites, are composed of a negatively charged nanolayer and a hydrated cation that balances the anion in the layer and has a high specific surface, mechanical characteristics, adsorption, and optical properties [80]. They are widely used in nanostructured platforms in health-related fields such as tissue engineering, regenerative medicine, and nano-drugs delivery systems [81].

Halloysite nanotubes (HNTs), a tubular clay nanomaterial, are the preferred choice of nanoclays for biopharmaceutical applications due to their cheap cost and non-toxicity. Currently, HNTs are often surface-modified to enhance their biocompatibility and hydrophilicity [82]. Chitosan (CTS)-functionalized HNTs have been used as responsive nano-supports for grafting copper (Cu) and laccase (Lac), which dramatically improves degradation resistance [47]. Utilizing HNT functionalized with 3-aminopropyltriethoxysilane (APTES-HNT) and endowed with surface aminopropyl may significantly enhance the adsorption capacity of HNT on cations and provide a nanoplatform for heavy metal wastewater treatment [48]. In addition, the tubular, hollow structure of HNT makes it a promising drug carrier. The strong interaction with the iron of APTES-HNT as a nanocarrier for ciprofloxacin (CIP) lowers CIP complexation with iron and promotes CIP bioavailability [49]. Embedding microparticles (MPs) with pH-responsive macropores in amine-functionalized HNTs might result in a high loading capacity, improved drug stability throughout encapsulation and gastrointestinal conditions, and effective medicine release [50]. Paclitaxel, an anticancer drug, is encapsulated in the tube, and the pH-responsive polymer poly (methacrylic acid-co-methyl methacrylate) is coated on the HNTs. It is feasible to prevent the depletion of paclitaxel in the gastrointestinal environment and assist in targeting the medication [51]. In recent years, a substantial amount of research has been conducted on HNTs as drug delivery platforms. However, the gut contains a great deal of unexplored potential for research. For instance, HNTs have the potential to be employed as nanoscaffolds to promote the proliferation and differentiation of intestinal epithelial cells. By modifying and encapsulating ROS-scavenging bioactive compounds to target sites of inflammation, HNTs can be used in a simple and low-cost ion-exchange procedure with metals with antimicrobial properties to regulate intestinal flora.

### Mechanisms of drug delivery by nanomaterials

After nanomaterials reach their target cells, they undergo a range of internalization processes, including pre-absorption, uptake, and transport [82, 83]. Nanomaterials may penetrate intestinal epithelial cells through endocytosis, where they are absorbed and used by the host. Endocytosis comprises phagocytic membrane invagination and intracellular vesicle formation [85, 86]; nevertheless, non-specific nanomaterials have a brief residence period in cells owing to the activation of cell efflux pumps triggered by high or low internal and external drug concentrations [87]. In phagocytosis with specific receptor mediation, large nanoparticle aggregates of nanomaterials are taken up into the interior of particular cells of macrophages, dendritic cells and M cells [88, 89]. The macrophages, dendritic cells and M cells at the severe mucosal damage site are exposed because of the loss of epithelial cells and intercellular junction proteins. In contrast, pinocytosis includes the reception of fluids and solutes without the participation of specialized receptors or cells. Cells internalize mixtures of nanomaterials and liquids through pinocytosis, which is reliant on actin motility and involves actin expansion and cholesterol elongation [89, 90]. According to specific research, nanoparticles enter cells primarily by clathrin-mediated endocytosis [91], while when nanomaterials are treated with chemicals like folic acid or cholesterol, nanomaterials are mostly internalized into the cell through vesicle protein-mediated endocytosis [91]. In addition, nanomaterial-mediated receptor recognition is widely used in cancer therapy. For example, nanomaterials with immune checkpoint inhibitor composites exhibit more robust targeting of immune checkpoint receptors on the surface of tumor cells [92]. In particular, nanoparticles encapsulated with some chemotherapeutic drugs, such as Adriamycin [93], specifically target cancer cells and reduce the cytotoxicity of chemotherapeutic drugs and systemic immunosuppression.

In conclusion, the development of nanomedicine based on nanomaterials is a valuable and promising endeavor. Different nanomaterials can serve as drug carriers for the detection and management of illnesses, provide a variety of accurate and speedy diagnostic instruments, and accomplish precise therapy through the nanometre platform [93]. Metal-based nanoparticles can be used to change the surface of hydrogels, nanofibers, and nanoclays to give them new functions and achieve the objective of antibacterial therapy in the intestine. Nanogels and nanoclays are typically naturally occurring, non-toxic, and harmless; have inherent cell- or tissue-targeting effects (e.g., hyaluronic acid can regulate macrophages [94] and montmorillonite can target damaged mucosa [95]); and are pH resistant, making them superior carriers for oral intestinal drugs to avoid the digestive effects of gastric juices. The mechanical strength of nanocomposite fibres enables their use as drug carriers, while their exceptional support properties enable in vitro examinations of the digestive system and aid in cell or tissue regeneration in vitro as nanoscaffolds. However, due to their nanoscale size, shape, exposure intensity, and human features, nanomaterials may cause tissue cell damage [96]. Specifically, the colon is the most remote section of the digestive system, and thus the selection of carriers must take into account the materials' safety, tolerability, and rapid release. Extensive testing is still required to identify the most effective nanomaterials for the treatment of intestinal disorders.

## Intestinal in vitro research boosted by nanomaterials

Nanomaterials are classified morphologically into four dimensions ranging from zero to three, of which three-dimensional nanomaterials have been a research hotspot in recent years. 3D printing is a bottom-up discrete-cumulative process based on a mathematical computer model [97]. With an accurately regulated shape and size, 3D printing of nanomaterials can imitate the multi-level structures seen in real tissues. It is a novel approach of manufacturing tissue scaffolds for use in tissue regeneration technologies and individualized treatment [98]. For some diseases, such as tumors, in particular, 3D cellular models (e.g., tumor spheroids) can mimic the drug response of primary human tumors, as well as in vitro studies of tumor physiology such as metabolic and chemical gradients, hypoxic environments, and cell–cell and cell–matrix interactions [99].

### Approaches of building 3D architecture in vitro

Currently, bioinks made of natural or synthetic materials may be utilized in 3D bioprinting. However, the primary demand is that its viscosity and shear thinning viscoplastic rheology should meet the application technology requirements to ensure biocompatibility and cell viability during printing. There are four primary kinds of bioprinting technologies that are widely accepted nowadays: inkjet-based, direct laser writing, extrusion-based, and photocuring-based [100]. Inkjet-based 3D employs a piezoelectric or thermally driven nozzle to split the bioink into micro-droplets, which may be printed in layers to construct a 3D structure containing cells [101]. Laser direct writing 3D bioprinting utilizes a substrate with equally dispersed laser absorbing material and bioink. During the process of laser irradiation, bubbles are formed, and as the bubbles expand, the bioink is propelled away from the substrate and onto the forming platform, where it is eventually propelled into a 3D shape by the 3D motion platform [102]. Extrusion-based 3D bioprinting is the most widely used bioprinting method for printing highly viscous biomaterials. This method uses a pneumatic or mechanically driven nozzle to extrude the bioink in a controlled manner, depositing it onto a platform to form a two-dimensional structure that builds up in layers as it moves in the "Z" direction to form a three-dimensional structure [103]. Photocuring-based 3D bioprinting utilizes a digital light projector to harden the whole surface of the bioink, which is more efficient and accurate regardless of the intricacy of the single-layer structure [104]. Even though each printing technique has its own benefits, it is vital to choose the most suitable printing process for the bioink based on its properties to create the most accurate biological model.

### Remodeling the intestinal microenvironment

**Fig. 3:**
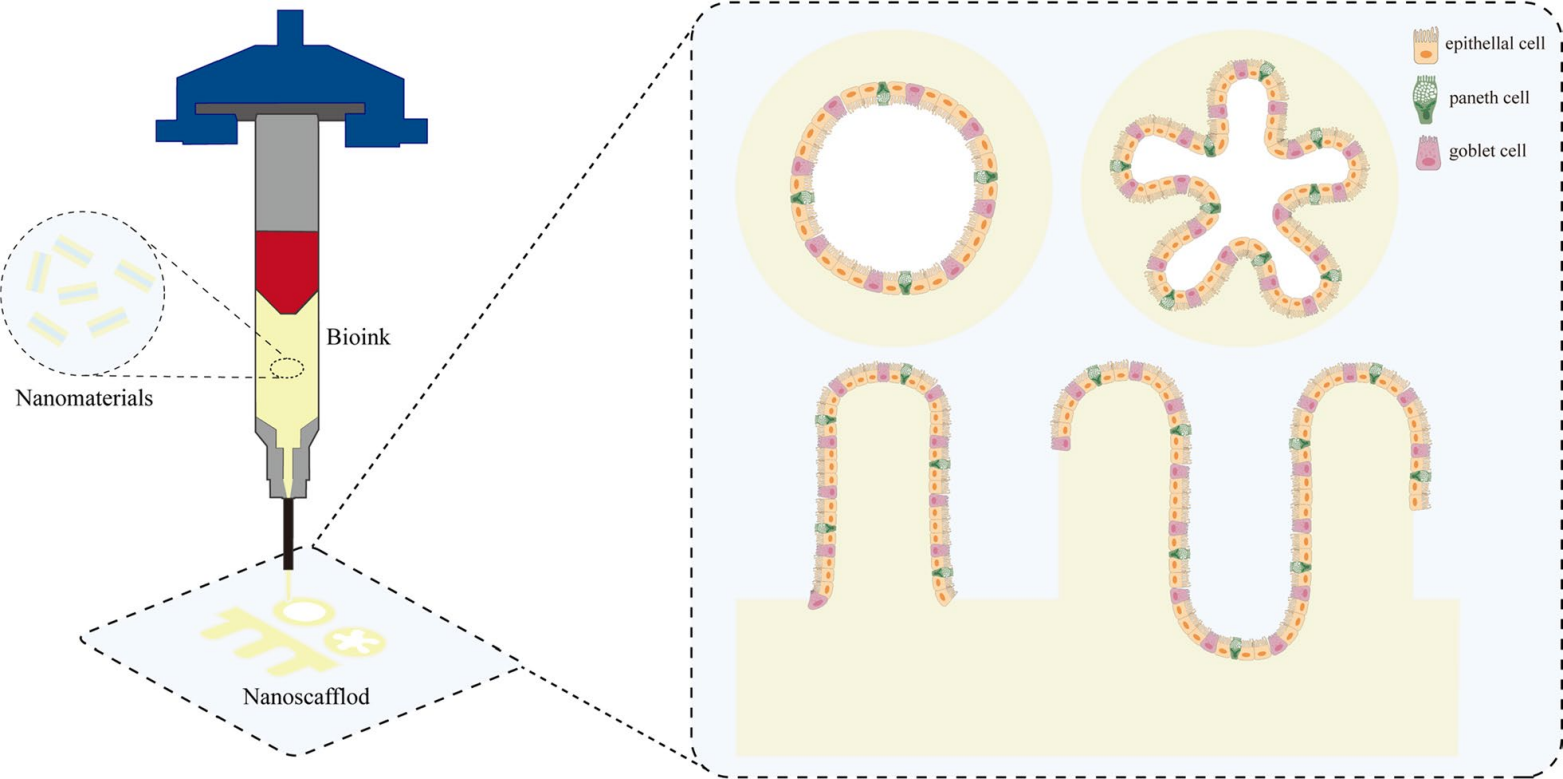
Different types of in vitro 3D architectures. **A** The prepared bioink is fed into the 3D printing system's nozzle at a predetermined flow rate, and the printing solution is charged by applying high voltage electricity to the needle's tip. Various forms of nanoscaffolds may be generated as required by adjusting the movement of the collecting platform. **B** Enterocytes are cultivated in a standard medium first, and then inoculated onto a nanoscaffold. Intestinal organoids create with the assistance of the nanoscaffold's spatial structure exhibit a range of structural characteristics, including spherical structures, budding structures, and complicated three-dimensional structures such as crypt or villi

The intestine is a marvelous area where intestinal cells coexist in harmony with symbiotic microbes in the lumen. The gut epithelium exhibits a characteristic architecture with high aspect ratio villus and crypt compartments, while outside the layer, a mucus layer rich in alpha-defensins, lysozyme, immunoglobulins, phospholipases, and nucleases in response to the luminal bacteria [105]. Simultaneously, ZO, JAM-A, Occludin, and other tight junction proteins act as a glue that holds intestinal epithelial cells together and forms a tight mucosal barrier [106]. Nonetheless, once the intestinal epithelial cells are injured, and the mucus layer and tight junction proteins are eliminated, this balance will be broken, resulting in damage to intestinal function. Commensal microorganisms may breach the barrier and enter the intestinal tissues or bloodstream, producing more severe intestinal inflammation [107]. Because of the complexity of the regulatory systems, studying intestinal processes, particularly in vitro, is more complicated. The limited culture period for traditional in vitro primary intestinal epithelial cell culture restricts our study on intestinal cell differentiation and the regulatory processes. However, when using 3D scaffolds made of nanomaterials, intestinal epithelial cells and mesenchymal cells grow for extended periods, proliferate and differentiate between mimicking the morphological characteristics and functions of epithelial cells, thereby restoring the cellular microenvironment in vivo (Fig. 3) [108]

Organoids have proven to be a universal means of studying intestinal health. When cells are co-cultured on 3D material scaffolds, a series of different 2D structures have been used to explore the effect of scaffolds with different structural features on intestinal cells. Cells' microenvironment is critical for reestablishing their shape and function. When the intestinal cells' microenvironment is similar to that of the body, their in vitro properties will be identical to those of the intestine in vivo [109]. L-N-isopropylacrylamide (pNIPAM), a non-biodegradable nanohydrogel system, is used to construct a 3D intestinal scaffold. After culture inoculated with Caco-2, it is found that the hydrated space of L-pNIPAM promotes the diffusion of nutrients and metabolites and stimulates extracellular matrix production. Surprisingly, it encourages Caco-2 cells (i.e., producing E-cadherin-actin complexes) to adhere the scaffold, forming comprehensive multi-layer cell clusters with microvilli brush borders [110]. Apart from the villi, crypt structures promote continuous epithelial cell turnover to support the maintenance of tissue integrity and function. Intestinal scaffolds are fabricated by high-resolution stereolithography (SLA) 3D printing by adding acrylic acid to poly (ethylene) glycol diacrylate (PEG-DA) to form a composite hydrogel network of negatively charged free carboxyl groups as a bioink. Unexpectedly, this process reproduces the crypt structure and size of the mouse intestinal epithelium and achieves complete colonization of Caco-2 cells from the crypt base to the top of the villus. At the same time, the hydrogel is not degraded by the cells [111]. The combination of organoids with nanomaterials is intriguing because it takes use of nanomaterials' capabilities in culture to complement organoids. According to the latest related research, the introduction of carbon nanotubes in a 3D model within an intestinal organoid increased the number of Paneth cells, enteroendocrine cells, goblet cells and absorptive enterocytes forming mature crypt-villi lumen structures. On the one hand, carbon nanotubes activate the Piezo-p38 MAPK-YAP signaling pathway via the release of metalloproteinases to alter the viscoelasticity of the extracellular matrix, promoting cell proliferation and differentiation. On the other hand, they upregulate the intracellular metabolic activity and respiration of mitochondria to promote cell groeth with an increasing production of ATP [112]. Nanomaterials are also applied in the in vitro modeling of intestinal cancer. Three heterogeneous cell populations (epithelial colon cancer cells, human intestinal fibroblasts, and human monocytes) are used to construct 3D models of multicellular tumor spheroids. The spermine-modified dextran acetal nano-drugs loaded with chemotherapeutic drug Nutlin-3a and colony-stimulating factor (GM-CSF) were tested on this model, which provides a therapeutic cellular model for prompting its translation to the clinic with reliable and predictive in vivo [113]. Thus, the cubic space of the 3D model of the intestinal epithelium promotes the continuous renewal of cells to support the maintenance of the integrity and function of the in vitro tissue model. Benefiting from the flexibility of 3D bioprinting technology, the fabrication of an in vitro platform with the subcellular structural resolution is beneficial for studying the fundamental mechanisms driving intestinal homeostasis and regeneration. These models facilitate basic research and provide a standardized platform for drug testing.

**Fig. 4:**
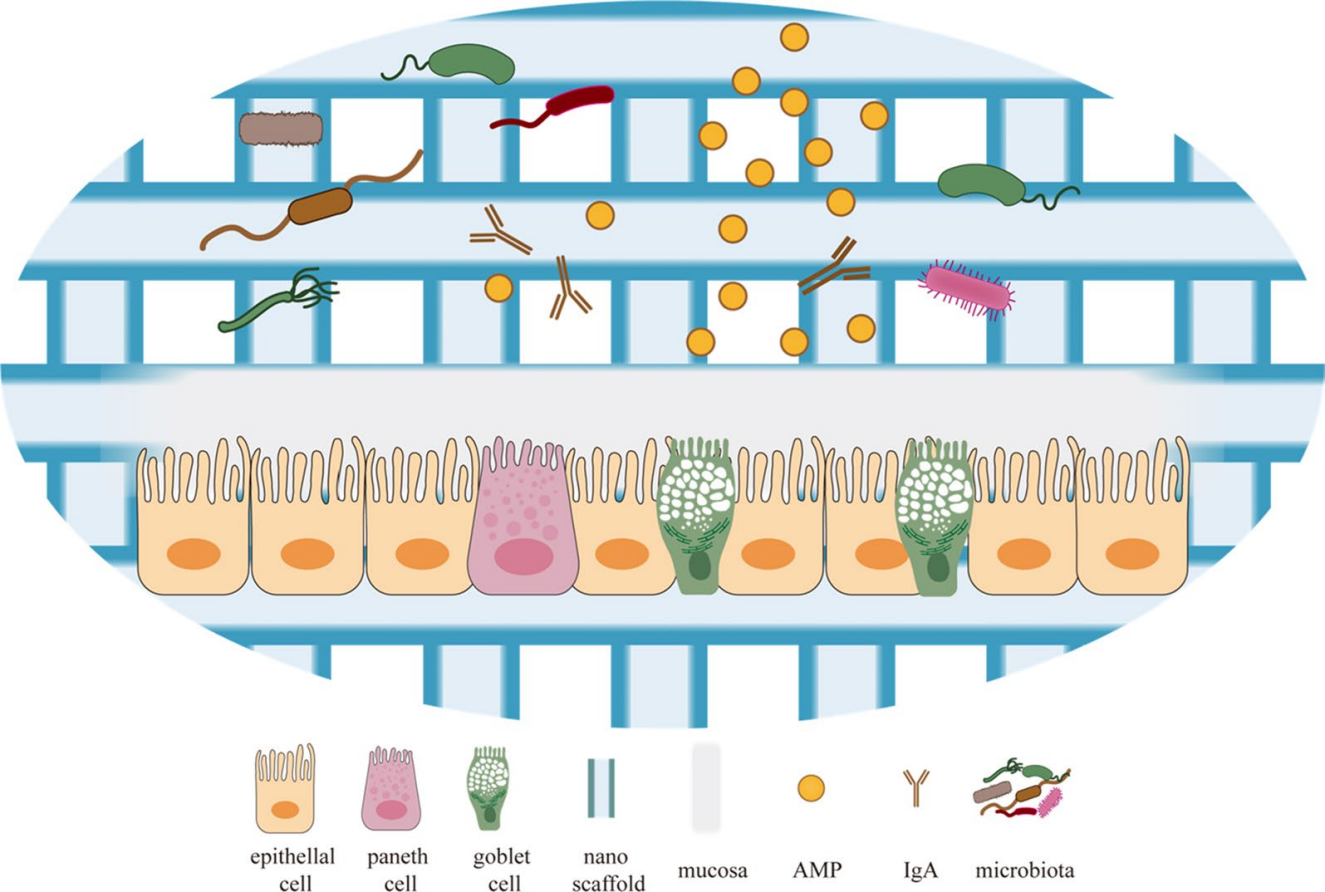
Remodeling the intestinal microenvironment. Using a 3D bioprinting method, nanofibers or other materials are employed to produce nanoscaffolds in vitro. After that, intestinal epithelial cells are seeded onto the culturing scaffolds. The nanoscaffold's three-dimensional space enables cells to adhere, proliferate, and differentiate. Under the activation of specific factors, they further differentiate into specialized cells such as paneth cells, goblet cells, and so on, which collectively form the intestinal epithelial structure. The nanoscaffold's multidimensional space is used to enable microbial survival. In vitro, nanoscaffolds re-establish spatial and temporal connections between intestinal epithelial cells and microbes

3D in vitro modeling has the potential to redesign the microenvironment of intestinal cells and re-establish the connections between intestinal microorganisms and intestinal cells (Fig. 4). An electrospun gelatin 3D scaffold can assist and guarantee the transplantation and growth of fecal microbiota. 16S rRNA reveals that this type of scaffold retains all of the phyla present in the fecal sample and the biodiversity and richness of the bacterial consortia. Meanwhile, the scaffold is maintained constant [114]. Creating an in vitro model of the gut microenvironment with controlled topography is a significant challenge in replicating the interaction between the gut microbiota and intestinal epithelial cells. For instance, the contradiction between the anaerobic environment of intestinal microbiota and the aerobic environment of epithelial cells is a point that cannot be ignored in the in vitro remodeling of the intestinal microenvironment. A novel intestinal microarray has been used to mimic the oxygen gradient of the intestinal epithelium under physiological conditions, enabling stable co-culture of the intestinal epithelium with complex intestinal flora, hence enabling better maintenance of bacterial diversity and structure [115]. However, the intestinal epithelial cells of this chip are functionally homogeneous and fail to differentiate to form a stable and mature villi-crypt structure.

## Nanomaterials for intestinal diagnosis and treatment in vivo

Due to metabolic and homeostatic disorders, chronic inflammation, fibrosis, and tumors in the gut threaten public health. In recent years, the associated disease susceptibility and pathogenesis have been hot research topics. Due to the lack of proper diagnosis and the optimal treatment at an early stage, intestinal cancer is often overlooked until symptoms appear at a later stage [116]. The development of nanomedicine has made the diagnosis and treatment of intestinal diseases possible. This emerging technology mainly uses the tiny size of nanomaterials to build drug delivery systems, avoiding the first-pass metabolism of the liver to deliver medicaments and directly targeting the diseased intestinal tract. It has dramatically improved the diagnostic and therapeutic effects [117].

### Medical diagnosis

**Fig. 5:**
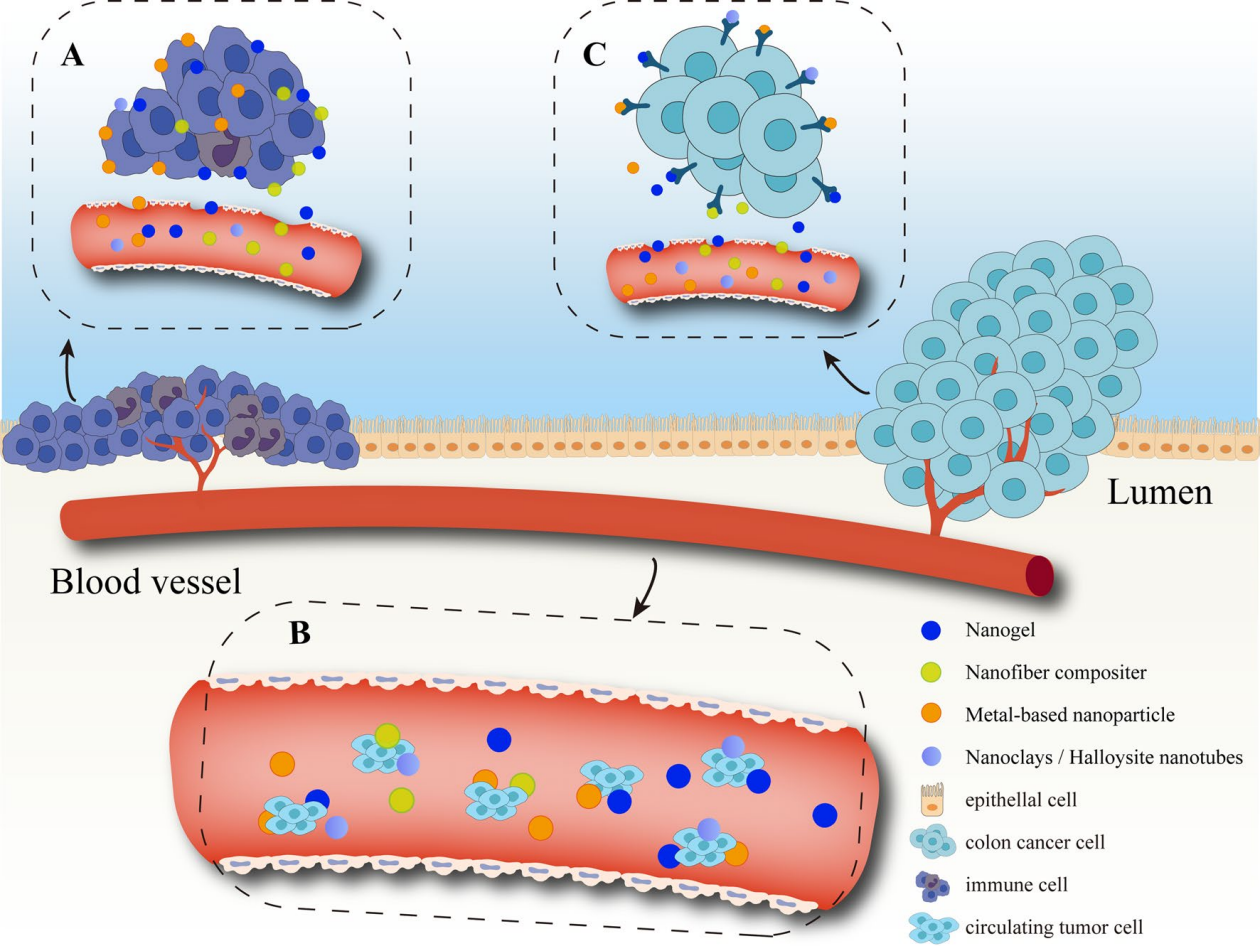
Several medical diagnostic pathways of nanomaterials on intestinal diseases. **A** The EPR effect is formed by the increased permeability of the vascular epithelial cells at the site of inflammation, and the nanomaterials can be targeted for precise detection. **B** Highly sensitive nanomaterials detect circulating cancer cells in the blood of cancer patients. **C** Nanomaterials specific detection of tumor cell surface markers

The development of medical diagnostics relying on nanomaterials is still being explored in depth. Cancer and inflammatory disorders are the primary concern aspects of nanomedical diagnostics, especially in the intestinal tract (Fig. 5). A significant feature of inflammatory bowel disease is damage to the mucosal epithelial barrier and increased barrier permeability. Due to the enhanced permeability and retention effect, tiny volume of nanomaterials is passively diffused to the inflamed area through leaky fenestrated capillaries [118]. This property has been taken advantage of in a study in which an analytical method for continuous fluorescence recovery after photobleaching was established. Inert fluorescently labeled nanoscale dextran is used to detect barrier permeability of the intestine and blood vessels in vivo. The continuous distribution of the probe is measured using fluorescence recovery after photobleaching. Dextran is found to have entered the circulation 7 h after lipopolysaccharide injection, while the plasma of PBS-treated mice is in trace amounts [119]. Nanomaterials have also been utilized to special label glucose in vivo to diagnose gestational diabetes mellitus [120]. In cancer patients, tumor cells spontaneously invade into the tissues surrounding the primary tumor cells. They enter the blood circulation and lymphatic circulation system to form circulating tumor cells and translocate to distant tissues. These cells re-exude, colonize, and proliferate, thus forming metastatic foci [121]. Recent studies have verified the utility of circulating tumor cells in peripheral blood for early diagnosis of cancer and prediction of disease progression. Therefore, researchers have designed a microfluidic electrical device to identify circulating tumor cells in the blood by distinguishing and enriching them with highly conductive graphene nanoplates. This enrichment efficiency reached nearly 500 times, with a recognition efficiency of 94% [122]. Besides, cancer cells are in an active state of high division and overexpress various surface antigens and receptors. The unique antigen receptor markers of cancer cells can also be helpful for early screening and diagnosis [123]. A tumor-targeted nanoparticle of polyethylene glycol-coupled hyaluronic acid has been synthesized that specifically binds to the overexpressed hyaluronic acid receptor on the surface of cancer cells. This nanoparticle accumulates selectively in colon cancer tissues after systemic administration, achieving early visualization and diagnosis of colon cancer by chemical coupling with near-infrared fluorescent dyes [124]. Likewise, in another study, tumor-targeting ligands such as peanut agglutinin, anti-carcinoembryonic antigen antibodies and tumor-associated glycoprotein-72 monoclonal antibodies are bioactivated by covalently binding protein nanoparticles carboxylic acid bonds, and specifically detect colon cancer tumors using NIR fluorescence [125].

### Precise treatment

Inflammatory bowel disease (IBD) is accompanied by systemic chronic inflammation and damage to the intestinal mucosal barrier, including ulcerative colitis and Crohn's disease [126]. Mucosal damage induces intestinal microbial disorder and immune response mechanisms dysfunction, further exacerbating IBD symptoms [127]. The latest developments in nanotechnology use a multidisciplinary approach to synthesize nanoparticles that can target specific cell subpopulations with a high degree of specificity, creating more possibilities for the precise treatment of IBD [128]. Consequently, the current intervention methods for IBD are mainly based on inflammation inhibition and mucosal repair.

#### Inflammation inhibition based on ROS scavenging

Inflammation suppression has been the dominant treatment modality for IBD. Nanomaterials are introduced to target the site of intestinal inflammation to suppress the immune response and relieve inflammation accurately. High levels of ROS are often present at sites of intestinal inflammation, and prolonged exposure to high levels of ROS can induce protein, lipid, and DNA damage, exacerbating mucosal damage, ulceration, and inflammation [129]. Clearing ROS to relieve inflammation is a route to treating IBD. Tungsten (W)-based polyoxometalate nanoclusters (W-POM NCs) have been shown to have excellent ROS scavenging ability. Orally administered W-POM NCs have excellent stability in acidic environments, scavenging excess ROS at inflammatory sites and restoring colonic microstructures, demonstrating the promising application on IBD [130]. Compared with natural enzymes, artificial enzymes based on nanomaterials are characterized by high stability and multiple catalytic active sites, and are gradually becoming alternatives to natural enzymes in various biomedical applications [131]. CeO_2_ is a multi-enzyme mimic for scavenging ROS and treating inflammatory illnesses because it has superoxide dismutase and catalase-like capabilities as well as hydroxyl radical scavenging capability. CeO_2_@MMT is obtained by in situ growth of cerium oxide nanoparticles (CeO_2_ NPs) on sheets of MMT, which directly targets the inflamed colonic segment under electrostatic action after oral administration. CeO_2_ NPs exert antioxidant effects to effectively scavenge ROS, significantly reducing inflammation at the lesion site and promoting anti-inflammatory immune response. The negatively charged MMT is well-tolerated, coated in the colon, not digested or absorbed, and expelled with CeO_2_ nanoparticles to decrease toxicity. ROS-catalyzing 2D vanadium carbide (V2C) MXene nanoenzymes contain inherent multienzyme-like activities, including superoxide dismutase, catalase, peroxidase, thiol peroxidase, glutathione peroxide, and haloperoxidase. Through the synergistic catalysis of multifunctional MXenzymes [132], intelligent cytoprotection against oxidative stress-induced inflammation and neurotoxicity may be accomplished. Taking advantage of the rare active N-group scavenging ability of rhodium nanoparticles and modified with polyethylene glycol to improve biocompatibility, the synthesized nanozyme show excellent intestinal inflammatory ROS scavenging ability [133]. Given the above, it is prospective to develop artificial enzymes with excellent redox properties and apply them to the treatment of intestinal inflammation.

#### Inflammation suppression based on immune cell regulation

The regression of intestinal inflammation is a complex process in which specific molecules and cells are involved, among which macrophages are gatekeepers in intestinal immune homeostasis and are considered essential contributors to the establishment and maintenance of intestinal homeostasis. Dysfunction of intestinal macrophages is thought to underlie the induction of chronic inflammation [134]. Therefore, understanding and regulating the mechanisms of differentiation and protection of intestinal macrophages is essential for intestinal inflammation suppression. It has been demonstrated that in the intestine of IBD patients, macrophages, as antigen-presenting cells secrete large amounts of *TNF-α* to regulate the secretion, migration, and activation of other immune cells or cytokines involved in the inflammatory response [135]. CDs/Man-NPs, a new nanocomposite material, may be used as a macrophage-targeted drug delivery carrier because it combines mannose nanoparticles with carbon dots (CDs/Man-NPs) through covalent conjugation, making it easier for inflammation-activated macrophages to ingest. *TNF-α*, *IL-8* proinflammatory factor expression is downregulated after accumulation in inflamed colonic sites [136]. Precise modulation of the tumor microenvironment is an effective way to treat colorectal cancer. Biocompatible non-covalent channel-based nanoparticles fabricated by host–guest complexation and self-assembly of mannose-modified-cyclodextrin with regorafenib (RG@M-γ-CD CNPs), reduce tumor cell survival, lesion angiogenesis, attenuate inflammation, and decrease tumor-associated macrophages activation [137]. By in situ mineralization covered with CaCO_3_ onto a hollow mesoporous Cu_2_O shell and wrapped with a layer of hyaluronic acid to create Cu_2_O@CaCO_3_@HA, another nanocomposite targeting the H_2_S-rich colorectal cancer tumor environment has been synthesized (CCH).CCH effectively neutralizes the tumor's acidic environment. More interestingly, CCH-based synergistic therapy can be achieved by reprogramming TAM from a phenotype that promotes tumor growth to one that kills tumors in patients with cancer [138]. The 12-mer D-enantiomeric peptide ^D^PMI^β^ is grafted onto Apamin using an unique "mirror image peptide grafting" approach, resulting in a chiral protein superparticle (^D^MSN) with exceptional cell membrane penetration and endosomal escape in vitro and in vivo. Moreover, ^D^MSN displayed p53-dependent anti-proliferative action and improved anti-programmed cell death protein (PD)-1 therapy, making colorectal treatment more clinically applicable [139]. Nucleotide drug delivery systems are a direction for nanomedicine preparation. Cationic polysaccharides can bind DNA to form stable nanoscale particles that can effectively deliver DNA to macrophages [140]. On this basis, a chitosan-alginate hydrogel drug delivery system containing triple helix *β*-glucanand polydeoxyadenylic acid [s-LNT/poly(dA)-DNA] is synthesized. Specific release into the inflamed intestine and internalization by immune cells such as macrophages were found after oral administration. s-LNT/poly(dA)-DNA effectively inhibited *TNF-α* expression, demonstrating the feasibility of treating inflammatory diseases [141]. Despite the fact that this sort of therapy, which depends on inflammatory regulatory systems, may offer patients with considerable relief, it requires a lifetime of medicine.

#### Mucosal repair

**Fig. 6:**
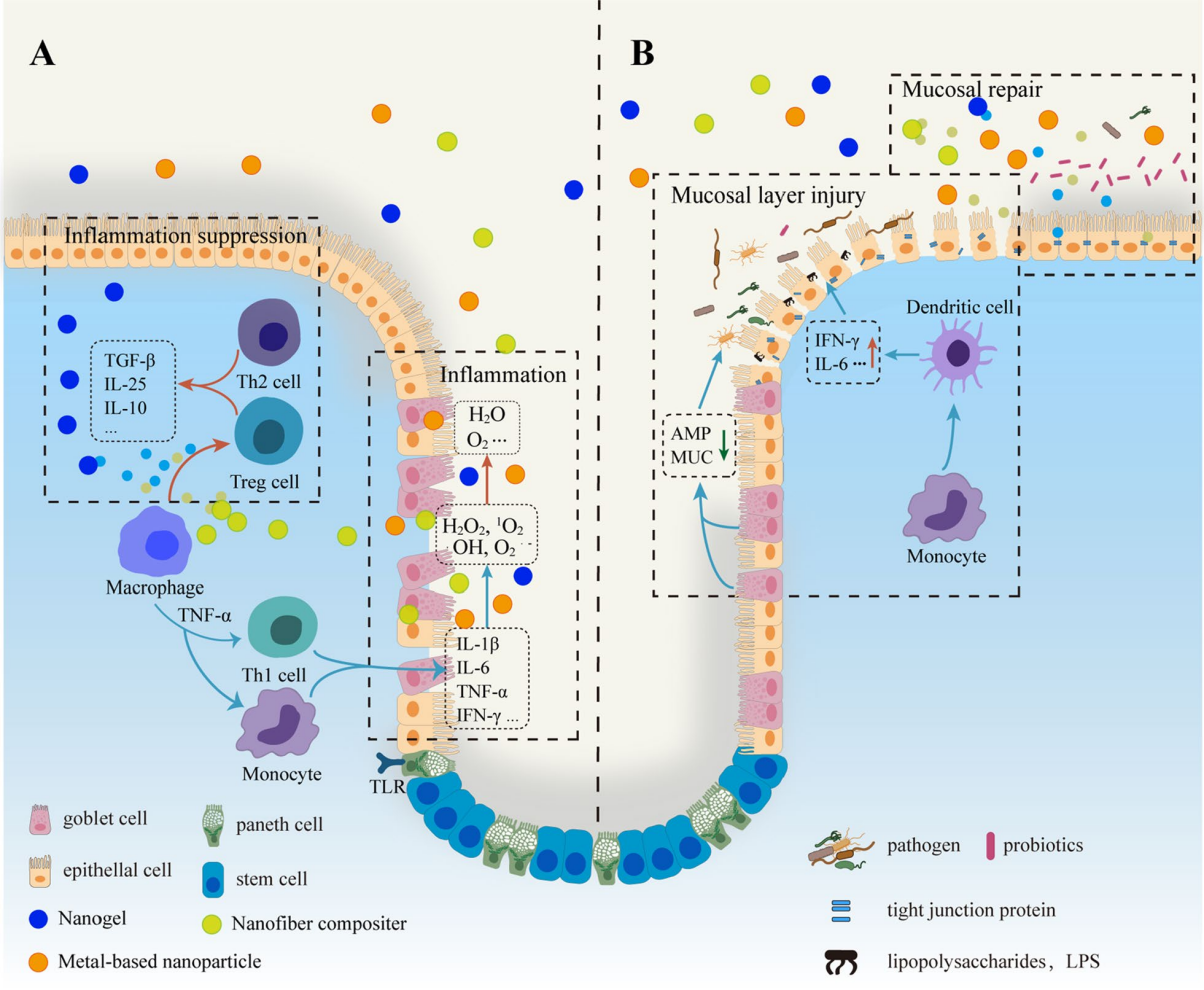
Therapeutic mechanisms of nanomaterials in the intestine. **A** In the intestine of IBD patients, inflammatory regions accumulate large amounts of ROS produced by goblet cells (H_2_O_2_, O_2_^·**−**^, ·OH, ^1^O_2_). This signal activates Toll-like receptor-mediated *NF-κB* signaling pathway. At the same time, macrophages produce large amounts of *TNF-α* and activate more monocytes as well as Th1 cells, resulting in large levels of pro-inflammatory factors *IL-1β*, *IL-6*, *IFN-γ*, *TNF-α*. Nanomaterials with antioxidant effect remove overexpressed ROS and convert them into water and oxygen. Meanwhile, the nano drug delivery system can release anti-inflammatory drugs in a targeted position, which inhibit *TNF-α* expression and induce Treg cells, Th2 cells to secrete *IL-10*, *IL-25*, *TGF-β* and other anti-inflammatory factors to relieve inflammation. **B** Mucosal damage is a sign of the intestinal tract in IBD patients. When goblet cells secrete insufficient amounts of AMP, MUC, the mucosal layer border is thinned or gapped. Pathogenic bacteria and their product LPS cross the mucosal barrier to induce an innate immune response. Immune cells such as dendritic cells produce pro-inflammatory factors IFN-γ and IL-6 in response to the inflammatory environment, resulting in mislocalization and reduced expression of tight junction proteins, further weakening the function of the intestinal mucosal barrier. Related functional nanomaterials or drug delivery systems increase the relative abundance of probiotics and contribute to the recovery of the mucosa and mucus layer by upregulating the expression of tight junction proteins (ZO-1, Occludin, Claudin). *IL-1β* interleukin-1β, *IL-6* interleukin-6, *ΝF-κB* nuclear factor-κB, *TNF-α* tumour necrosis factor α, *IL-10* interleukin-10, *IL-25* interleukin-25, *AMP* antimicrobial peptide, *MUC* mucin, *IFN-γ* interferon-γ, *TGF-β* transforming growth factor-β

Mucosal repair is the fundamental purpose of treating inflammatory bowel disease. Intestinal mucosa repair essentially refers to the spatiotemporal interaction of soluble mediators with intestinal epithelial cells, immune cells, microbiota, and other cells of the lamina propria, achieving the restoration of selective permeability of the mucosal layer and the complex and dynamic association with immune cells [142, 143]. In a recent study, the amphiphilic conjugate of hyaluronic acid-bilirubin nano-drugs (HABN), precisely targeting DSS-injured colonic sites, accumulates anti-inflammatory macrophages upregulate the expression levels of tight junction proteins and antimicrobial peptides in DSS-injured colonic epithelial cells to promote recovery of the mucosa and mucus layer. HABN alter the composition of the intestinal flora, increasing the diversity and relative abundance of *A. muciniphila* (promoting mucus tight junction protein production and expression), *Clostridium* XIVα, and *Lactobacillus* (colonic anti-inflammatory). The gut-targeting properties of hyaluronic acid have also been used as a probiotic delivery. An HA-SH self-crossing hydrogel is used for targeted delivery of *Lactobacillus rhamnosus* to address the loss of probiotic bacteria in quantity due to oral administration. It is also sensitive to H_2_S-producing pathogenic bacteria. It competes competitively with pathogens for binding sites to prevent the attachment of pathogenic bacteria in the host intestine, thereby improving mucosal and intestinal flora and alleviating bacterial colonic inflammation. The natural active ingredient has an excellent therapeutic effect on IBD but has disadvantages of poor solubility, poor gastrointestinal stability, rapid metabolism, and fast systemic clearance [144]. Chondroitol is encapsulated in core–shell nanoparticles using maize alcohol-soluble protein and functionalized on the surface of the nanoparticles by chondroitin sulfate, a CD44 receptor activator. Following oral administration, it boosts colonic epithelial formation and protects colonic goblet cells [145]. Nucleic acid drugs with multi functionalities are also used for the mucosal repair in ulcerative colitis. miR-320 is a model nucleic acid drug made using polymeric nanocapsules and alginate. The nanomedicine rapidly passes through the small intestine, relying on enzymes produced by bacteria in the colon to break down alginate and release miR-320. This non-coding RNA regulates colonic epithelial cells and submucosal macrophages, modulating the inflammatory response and promoting mucosal healing [146]. The ultimate goal of both inflammation inhibition and mucosal layer repair is to restore the function of the intestinal mucosal barrier, to re-establish the close interaction between the mucosa and the microbiome, and to enhance tolerance and resistance to pathogens (Fig. 6).

Overall, studies of nano-drugs delivery systems have demonstrated the formulation of various nanomaterials for intestinal treatment. Synergistic treatment, whether with current small-molecule medications such as regorafenib, metformin [147], or with prospective therapies such as siRNA [148], the nano-drugs delivery technology significantly increases the therapeutic impact of the original drugs and has a promising clinical application outlook.

## Conclusions and perspectives

**Fig. 7:**
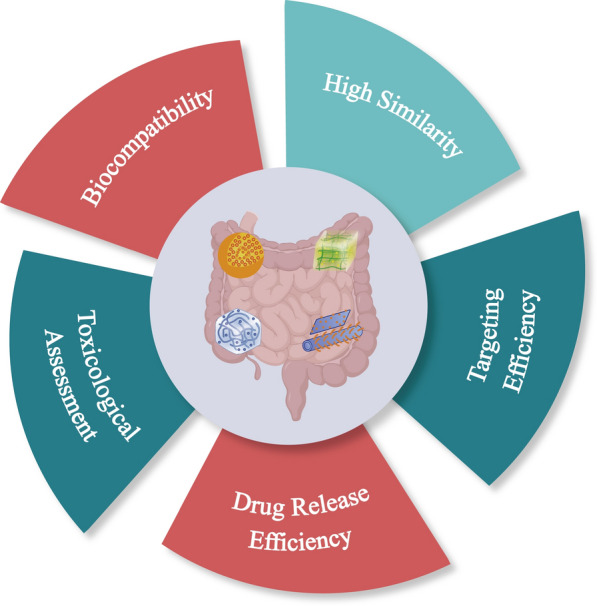
Challenges of nanomaterials in intestinal applications

The purpose of this review is to outline the current status of research on nanomaterials in intestinal in vitro modelling. We discuss the current situation and difficulties of using nanotechnology for the treatment of intestinal diseases with the point of entry of intestinal medicine diagnosis and precision treatment, and propose a future direction for nanoscaffolds in tissue organ regeneration technology. Nanoscaffolds provide a near in vivo environment for cells in vitro. They can also contribute to effective cell differentiation by adding or associating different materials to replicate an intestinal model in an in vitro environment that maintains a high uniformity with the in vivo intestinal tissue structure. Furthermore, the nanomaterial delivery system solves the drug off-target problem to a certain extent, reduces drug dosage and decreases side effects. Patent applications and clinical trials on nanomaterials for intestinal diagnosis and treatment have also yielded some results. For instance, a nanoparticle combining a hydrophilic component of basic amino acids, a hydrophobic portion of polysaccharides, and medicines for radiation protection of the small intestine, can effectively suppress ionizing radiation-induced cell death [149]. The mucosal adhesive nanoparticle delivery method composed of amphiphilic macromolecules (polylactic acid and dextran) exhibits a high loading ratio and gradual release at the mucosal site [150]. An oral formulation of negatively charged nanoenzyme derived from cerium oxide has been created to target positively charged regions of pathological enteritis to minimize local inflammation and mend ulcers [151]. Despite the considerable developments that have been achieved in these areas, some fundamental and essential issues still require more research attention (Fig. 7).

- The optimization of nanoscaffolds is extremely attractive, and its application in tissue organ regeneration offers excellent prospects. Compared to conventional allografts, it allows flexibility in designing the final morphology of cell proliferation and differentiation, reduces healing time and complications, and reduces immune rejection reactions induced by allografts. However, more efforts are still needed to improve and optimize nanoscaffolds in light of the following.
- Biocompatibility is a dynamic process that depends on the interaction between the material and the environment. It is a crucial factor in the selection of scaffold materials and an important indicator to be considered and evaluated for nanoscaffolds in biomedical applications. As it stands, no perfectly inert material can meet the requirements. Surface modification is an effective means to improve biocompatibility and currently relies on surface modification techniques to regulate the mechanical properties of materials, cell adhesion, reversible friction [152]. While efficiency, cost, stability and operability have been plagued by issues with the surface modification process. More importantly, surface multi-functionalization and even local surface functionalization need to be further explored.
- Maintenance of a high degree of similarity is required for the environmental patterns of the extracellular matrix. Nanoscaffolds for tissue engineering need to maintain morphological and functional similarity with in vivo organs and tissues when constructing organs or tissues in vitro. The in vitro construction of the gut has been stuck in single intestinal epithelial cell cultures or a simple co-culture. It is hypothesized that nanoscaffolds can be used to mimic the coexistence morphology of intestinal epithelial cells, immune cells, and intestinal microbiota in vivo. In this case, gut research will take off in leaps and bounds.
- In precision medicine, nanomaterials focus on diagnostics and drug development. Clinical diagnostic efficiency based on nanomaterial properties has greatly improved. At the same time, nanomedicine delivery systems provide an effective way to address significant diseases today, especially in oncology, immunotherapy, and genetic engineering. However, nano-precision medicine has many limitations, and the progress made in clinical practice is still relatively low. It is expected that more work will be needed in the future.
- One advantage of nanomedicines is targeting, especially in intestinal inflammation or colon cancer. Nanomedicines can be delivered directly to the site of action, thus reducing adverse effects and improving therapeutic efficiency. The strategy of using nanomedicines to target immune cells directly can modulate the immune response in patients with autoimmune diseases after organ transplantation. Although targeting offers great therapeutic convenience, limitations remain due to the multiplexed targeting performance of nanomaterials, the human body environment, and the immune system’s complexity [153].
- Nano-drugs delivery systems can ensure a higher and longer duration of drug bioavailability, thus improving therapeutic efficacy. The application of this strategy to intestinal diseases is of great interest, addressing the problems posed by the metabolism of intestinal drugs by digestive juices in the stomach and small intestine before transport to the lesion site. However, while nano-drugs delivery systems have the advantage of enhanced bioavailability, they are limited by control over the drug release rate. Excessively long or short drug release times do not provide good relief and may even lead to other adverse effects. Therefore, more efforts are still needed in this area.
- Toxicological evaluation is essential for the clinical use of nanomaterials. At present, nanomaterials’ diagnostic and therapeutic roles in diseases have been reported one after another, but few of them have been applied in a clinical setting. Most of the early toxicity studies of nanomaterials are limited to cell culture analysis, which only focuse on cell viability, and the effects on genome and proteome are not fully understood [154].

## Data Availability

Not applicable.
